# Oxide Formation during Transpassive Material Removal of Martensitic 42CrMo4 Steel by Electrochemical Machining

**DOI:** 10.3390/ma14020402

**Published:** 2021-01-15

**Authors:** Daniela Zander, Alexander Schupp, Oliver Beyss, Bob Rommes, Andreas Klink

**Affiliations:** 1 Chair of Corrosion and Corrosion Protection, Foundry Institute, RWTH Aachen University, Intzestr. 5, 52072 Aachen, Germany; a.schupp@gi.rwth-aachen.de (A.S.); o.beyss@gi.rwth-aachen.de (O.B.); 2 Laboratory for Machine Tools and Production Engineering (WZL), RWTH Aachen University, Campus-Boulevard 30, 52074 Aachen, Germany; b.rommes@wzl.rwth-aachen.de (B.R.); a.klink@wzl.rwth-aachen.de (A.K.)

**Keywords:** 42CrMo4 steel, electrochemical machining, rim zone modification, Fe_3−x_O_4_ mixed oxide, transpassive area

## Abstract

The efficiency of material removal by electrochemical machining (ECM) and rim zone modifications is highly dependent on material composition, the chemical surface condition at the break through potential, the electrolyte, the machining parameters and the resulting current densities and local current density distribution at the surfaces. The ECM process is mechanistically determined by transpassive anodic metal dissolution and layer formation at high voltages and specific electrolytic compositions. The mechanisms of transpassive anodic metal dissolution and oxide formation are not fully understood yet for steels such as 42CrMo4. Therefore, martensitic 42CrMo4 was subjected to ECM in sodium nitrate solution with two different current densities and compared to the native oxide of ground 42CrMo4. The material removal rate as well as anodic dissolution and transpassive oxide formation were investigated by mass spectroscopic analysis (ICP-MS) and (angle-resolved) X-ray photoelectron spectroscopy ((AR)XPS) after ECM. The results revealed the formation of a Fe_3−x_O_4_ mixed oxide and a change of the oxidation state for iron, chromium and molybdenum, e.g., 25% Fe (II) was present in the oxide at 20.6 A/cm^2^ and was substituted by Fe (III) at 34.0 A/cm^2^ to an amount of 10% Fe (II). Furthermore, ECM processing of 42CrMo4 in sodium nitrate solution was strongly determined by a stationary process with two parallel running steps: 1. Transpassive Fe_3−x_O_4_ mixed oxide formation/repassivation; as well as 2. dissolution of the transpassive oxide at the metal surface.

## 1. Introduction

Electrochemical machining (ECM) of metals is increasingly gaining in importance in the field of subtractive manufacturing technology. In recent years, intensive research has opened up new application areas for the ECM process, for example, the production of turbine blades [1,2], the machining of difficult to cut materials [3] and the production of biomechanical implants [4]. The most important advantage of ECM is that even difficult-to-cut materials can be machined at high material removal rates with almost no tool wear [5]. In addition, good surface finishing can be achieved, as shown by the example of 42CrMo4 steel. 42CrMo4 is mostly used for components like crankshafts [6,7]. It is usually machined with abrasive (e.g., grinding) or thermal processes (e.g., electric discharge machining). These abrasive or thermal processes lead to undesired changes in the rim zone, such as white layers, residual stresses, or heat-affected zones, which might have a negative effect on the functionality of the component [8,9,10]. Recent results after ECM in sodium nitrate revealed that processing and surface finishing by ECM does not lead to the formation of the previously mentioned changes in the rim zone. In addition, ECM enables the removal of these undesirable rim zone modifications and the adjustment of improved surface properties [8,11]. However, in addition to the advantages mentioned, the ECM process also shows disadvantages. First, it is not suitable for machining specific complex geometries. Second, a new tool must usually be made for new component geometries, which often makes the use of ECM cost-effective only for large production batches. Third, the disposal of the process electrolyte used can lead to problems, especially if it has accumulated heavy metals such as chromium or cadmium [5,12]. The ECM process is mechanistically determined by transpassive anodic metal dissolution and layer formation at high voltages and specific electrolytic compositions. Various mechanisms are assumed to explain the anodic material removal accompanied by a transpassive layer formation of iron and steels during the ECM process. The latter is strongly dependent on the material composition, the chemical surface condition at the break through potential, the electrolyte, the machining parameters, and the resulting current densities and local current density distribution at the surfaces.

It is well known that at the break through potential the transformation of the passive layer as well as oxygen evolution leads to a significant increase of the transpassive current densities. Numerous investigations have been carried out on the chemical composition and structure of the passive layer formed on iron between the passivation and breakthrough potential in borate buffer solution (pH 8.4), which is assumed to be comparable in terms of pH to the sodium nitrate solution used in this work. Based on the results by Nagayama et al. [13], the passive layer consists of superimposed oxides. Accordingly, a two-layer structure composed of Fe_3_O_4_ and γ-Fe_2_O_3_ was assumed. Davenport et al. [14] were able to investigate passive layers on iron in borate buffer solution using in situ X-ray absorption near-edge structure spectroscopy (XANES). In contrast to Nagayama et al. [13], the passive layer proposed was a continuous oxide. In this case, Fe_2_O_3_ was mainly present at the interface between the oxide and the electrolyte. With increasing thickness of the oxide, the proportion of Fe_3_O_4_ in the oxide increases continuously towards the oxide/metal interface. However, the question of whether the oxide was crystalline or amorphous was conclusively clarified in this study. Ryan et al. [15] confirmed the formation of crystalline γ-Fe_2_O_3_ at the oxide/electrolyte interface by using atomically resolved scanning tunneling microscopy (STM). In addition, the change of the oxidation state of the passive layer was also reported for highly alkaline electrolytes. XPS investigations by Haupt et al. [16] revealed the formation of an inner Fe (II) oxide and its transformation to Fe (III) with increasing potentials in 1 mol/l NaOH solution.

Considering the formation and transformation of surface layers during ECM processing of iron at transpassive current densities, Foley et al. [17] reported as early as 1967 on the formation of an oxide layer on the surface of iron for a borate buffer solution at low voltages of about 1–2 V, which is similar to a Fe_3_O_4_ spinel. The group of Rokosz, Hryniewicz et al. [18,19,20,21,22,23,24,25] analyzed the formation of oxide layers on different stainless steels containing chromium after electropolishing at low transpassive current densities of 0.5–10 A/cm^2^ in a mixture of phosphoric and sulfuric acid using XPS. It was proven that the thickness and the chemical composition of the oxide layer formed during the process depends on the current density, the material and the electrolyte. However, it was reported that the oxide layer consisted mainly of iron oxide, either in the oxidation state of Fe (II) or Fe (III), and was enriched by additional alloying elements, such as chromium or molybdenum, for acidic electrolytes at low transpassive current densities. Considering the oxide formation for iron at higher transpassive current densities and a sodium nitrate electrolyte, a layer consisting mainly of Fe_2_O_3_ with partially bivalent iron Fe (II) was observed as early as 1984 by Datta et al. [26] using atomic emission spectroscopy (AES) and XPS. The average thickness of the oxide layer was reported to be about 2.5–10 nm after ECM processing at 20 A/cm^2^ compared to a thickness of about 2–4.5 nm for the native oxide. In addition, it was observed that the thickness of the oxide layer strongly depended on the current density. An oxide layer with a maximum thickness of about 20 nm was observed at low current densities up to 8 A/cm^2^ and oxygen evolution was a predominant reaction. However, with increasing current densities above 8 A/cm^2^, the thickness of the oxide layer decreased again.

Additional microflow cell investigations on the oxide formation of iron in sodium nitrate solution by Lohrengel et al. [27,28,29,30] confirmed the formation of Fe_2_O_3_ at low transpassive current densities. These results were obtained by analyzing the oxidation state of iron within the electrolyte by UV-VIS. At current densities above about 10 A/cm^2^, however, a mixture of Fe_2_O_3_ and Fe_3_O_4_ seemed to be present. Furthermore, the proportion of Fe_3_O_4_ increased with increasing current densities. Wang et al. confirmed these results by XPS studies on stainless steel that was ECM processed in sodium nitrate solution [31]. In contrast, Münninghoff [32] did not detect any significant changes in the oxide layer composition using a microflow cell at current densities between 7–52 A/cm^2^ by analyzing the reaction products in the process electrolyte by UV-VIS. It was proposed that both oxides, i.e., Fe_2_O_3_ and Fe_3_O_4_, were formed at all investigated current densities.

The composition, structure and thickness of the oxide layer formed during the ECM process influence the efficiency of material removal, the achievable manufacturing accuracy and surface quality. According to Hoare [33], well-protecting passive layers, such as those that occur during machining of iron in sodium nitrate solution, slow down material removal. However, good surface finishes can be achieved. When machining iron in sodium chloride solution, on the other hand, no stable oxide layer can be formed. This leads to a strong increase in the material removal rate. However, geometrically precise material removal is almost impossible [33]. Bergs et al. [34] demonstrated the importance of an intact, protective oxide layer for the surface integrity obtained after ECM. It was shown that damage to the oxide layer on 42CrMo4 steel in sodium nitrate solution led to selective material removal in the ECM process and thus to an increase in surface roughness.

Besides the controversial observations on the formation of oxide layers during ECM processing of iron in sodium nitrate solution at high transpassive current densities, the formation of an additional viscous layer of hydrated iron nitrate has been reported by Lohrengel et al. [27,28,29,30], Moser et al. [35] and Rosenkranz [36]. It is assumed that the electrolyte on the surface is rapidly supersaturated with iron ions due to the strong iron dissolution and forms a polishing film. The iron nitrate polishing film is depleted in free water. Furthermore, the diffusion of water through the polishing film to the material surface is also inhibited, and is therefore assumed to be one important speed-determining step during the ECM process. The thickness of the iron nitrate film depends mainly on the current density during the ECM process and the flow conditions in the electrolyte. While an increase in current density leads to an increase in film thickness, an increased flow rate of the electrolyte reduces the film thickness. However, investigations of the formation of an additional polishing film at the oxide/electrolyte interface of either iron in sodium chloride solution [37] or other metals, such as copper, in sodium nitrate solution [38] or phosphoric acid [39] also revealed a film formation with different viscosities and composition.

In summary, the oxide layers that form in the transpassive area at low (e.g., electro polishing) and high (e.g., ECM) anodic current densities have come increasingly into the spotlight for predicting the electrochemical metal dissolution and material removal in recent years. However, determining the overall and local stoichiometric composition, the chemical distribution of additional alloying elements and structure of transpassive layers on ferrous materials is a challenge, and discussions have remained controversial for a long time. Therefore, the aim of this work is to investigate how electrochemical machining affects the composition, specifically the chemical distribution of the alloying elements, and structure, e.g., given by the oxidation state, of the oxide layer on a martensitic 42CrMo4 steel. Furthermore, the influence of the current density on the mechanism of oxide layer formation will be studied.

## 2. Materials and Methods

### 2.1. Materials

The formation of oxides during ECM was investigated for martensitic 42CrMo4 steel (AISI4140) surfaces. The main alloying elements were chromium, manganese and molybdenum. The detailed chemical composition of the steel is shown in Table 1.

The 42CrMo4 steel was austenitized for 2 h at 850 °C and quenched in oil at 60 °C. Afterwards, the material was tempered at 400 °C for 4 h and cooled down to room temperature in air. The 42CrMo4 in quenched and tempered condition had a hardness of 470 HV0.2, a Young’s modulus of 202,800 MPa, a yield strength of 1430 MPa and a tensile strength of 1570 MPa. Finally, the material was cut into square plates with an edge length of 10 mm and a height of 2 mm and ground with 2000 SiC abrasive paper to obtain comparable initial surface conditions.

### 2.2. Electrochemical Machining (ECM)

The martensitic 42CrMo4 was electrochemically machined using an EMAG PTS 1500 ECM device (EMAG GmbH & Co. KG, Salach, Germany) with an applied external DC voltage of 19 V. A sodium nitrate electrolyte with a conductivity of 135 mS/cm and a pH value of about 8 was used as a process electrolyte at a temperature of 36 °C. The flow rate of the electrolyte was 4.4 L/min. 42CrMo4 was used as the anode and a movable stainless steel performed as the cathode during the ECM process. Both anode and cathode exhibited a surface area of 1 cm^2^.

Two different ECM processes were performed on 42CrMo4: ECM-A and ECM-B. The two processes mainly differ in the size of the working gap between anode and cathode. Furthermore, the feed rate of the cathode was varied, as shown in Table 2. The process parameters were varied with the purpose of setting different current densities in the ECM process. During ECM, the current flow and the height decrease of the 42CrMo4 plates were measured.

### 2.3. Chemical Analysis of the Electrolyte before and after ECM

The process electrolyte was analyzed in terms of its chemical composition before and after ECM using mass spectroscopy with inductively coupled plasma (ICP-MS, NexION 2000, PerkinElmer LAS (Germany) GmbH, Rodgau, Germany). The advantage of this analysis is, on the one hand, to obtain information about the amount of material removed during ECM, and on the other hand, it makes it possible to draw conclusions on the material removal mechanism. Electrolyte samples were taken after reaching stationary ECM process conditions, which means for ECM-A after a period of 80 s and for ECM-B after a period of 10 s. Before ICP-MS measurements, the samples were diluted with 2% nitric acid in a ratio of 1 to 50.

### 2.4. Rim Zone Analysis of the Native Oxide and the Oxide after ECM Processing

X-ray photoelectron spectroscopy (XPS) analyses of the ground as well as ECM surfaces were carried out in a Kratos Axis Supra instrument (Kratos Analytical Ltd, Manchester, UK) using a monochromatic Al Kα X-ray source (hν = 1486.6 eV). High resolution spectra of the O 1s, C 1s, Fe 2p, Cr 2p, Mo 3d, and N 1s regions were obtained using a pass energy of 20 eV and a step size of 0.1 eV. Angle-resolved measurements were performed with take-off angles of 0°, 30° and 45° with respect to the surface normal. Peak deconvolution and quantification were performed using the ESCApe software provided by Kratos Analytical Ltd. (Manchester, UK). All spectra were charge corrected by assigning the adventitious C-C/C-H contamination component in the C 1s peak to 284.8 eV. Shirley type backgrounds were applied, and peak fitting was performed using Gauss-Lorentz (30/70) type line shapes with an exception for metallic Fe and Cr components, which required asymmetric line shapes.

## 3. Results

Material removal of 42CrMo4 in sodium nitrate solution by ECM is determined by metal dissolution, and the formation of an oxide layer and a polishing film at the surface. Further, it is well known that the current density significantly influences the material removal rate during ECM, and therefore the anodic dissolution and oxide layer formation, at high transpassive potentials. However, the influence of the current density on the mechanism of metal dissolution and the oxide formation at the surface is still controversial discussed [36,40].

**Figure 1:**
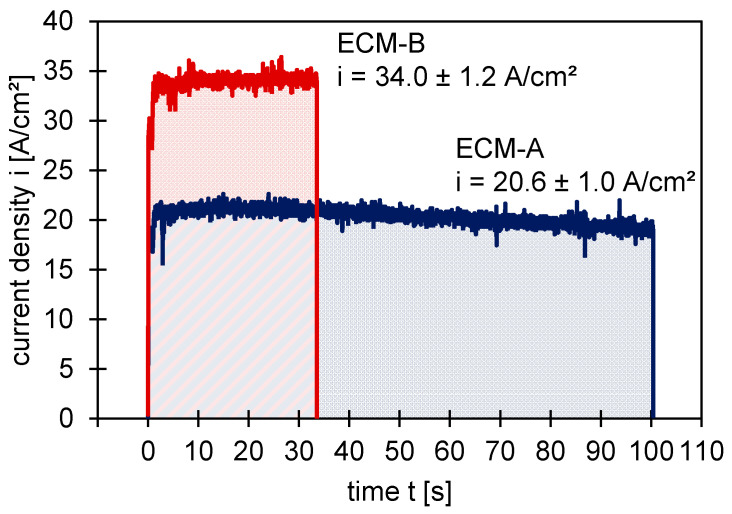
Current density during ECM of 42CrMo4 in 2.5 M sodium nitrate solution.

Focusing first on the influence of the current density on the electrochemical material removal, the current densities for both the ECM-A and ECM-B processes were determined. Figure 1 shows the course of the current density during the two ECM processes as a function of time. After a short, approximately three second stabilization phase, the current density is roughly constant for the duration of the remaining machining process. The average current density for the ECM-A process is 20.6 ± 1.0 A/cm^2^ and for the ECM-B process 34.0 ± 1.2 A/cm^2^.

The influence of the current density on the material removal and the efficiency of the ECM process was determined by three different methods: First, a theoretical material removal was calculated using Faraday’s law and considering the measured current densities. Second, the material removal was determined by measuring the height difference of the 42CrMo4 samples before and after ECM; and third, the material removal was calculated by analyzing the mass of the reaction products in the electrolyte with process time using ICP-MS. The results of the differently calculated material removal rates are compared and shown in Table 3.

The material removal rate of ECM-B is, independent of the measuring and calculation method, about twice as high as that of ECM-A. In addition, it is noticeable that the real removal rate is lower when calculating via height difference than when calculating via ICP-MS. This is due to the fact that in the height measurement only the highest point of the sample is measured. However, since the removal is not uniform over the entire surface, the real material removal is underestimated when calculating via height measurement. The calculation via ICP-MS measurement of the removal products in the electrolyte is therefore better suited to describe the real material removal rate. A further advantage is the increased accuracy of the ICP-MS measurements compared to height measurement. For all further calculations in this work, the removal rates of the ICP-MS measurement are used.

The efficiency of the ECM process can be determined by comparing the real material removal with the theoretical material removal (Faraday’s law). The charge yield η is a measure for the efficiency of material removal. It is calculated from the theoretical (*mr_theoretical_*) and the real measured material removal (*mr_real_*), as shown in Equation (1).

(1) $$\eta =\frac{mr_{real}}{mr_{theoretical}}$$

The charge yield of the ECM-B process (70%) is higher than that of the ECM-A process (60%). This means that the ECM-A process has relatively higher losses. Therefore, less energy is available for metal dissolution.

**Figure 2:**
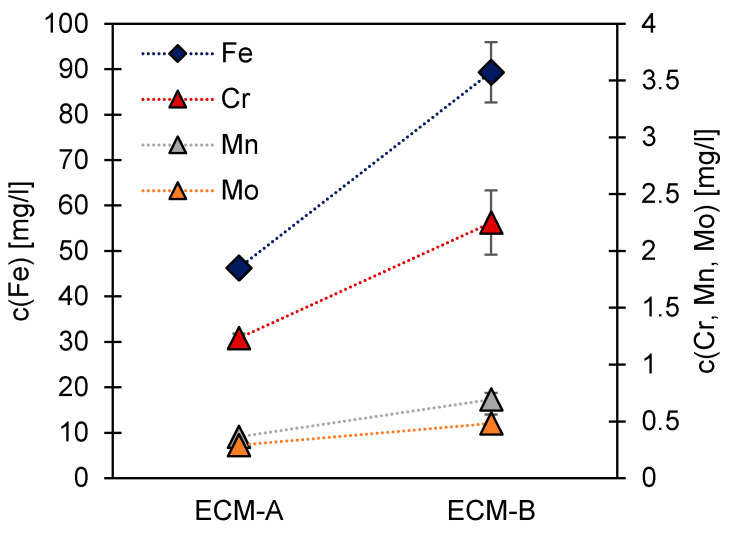
ICP-MS analysis of iron, chromium, manganese and molybdenum after ECM-A (20.6 A/cm^2^) and ECM-B (34.0 A/cm^2^) processing in sodium nitrate electrolyte.

For a detailed understanding of the anodic metal dissolution mechanisms dependent on the current densities, the composition of the process electrolyte was determined for iron, chromium, manganese and molybdenum by ICP-MS. Figure 2 reveals that during the ECM-A process, 46.3 mg/L iron, 1.2 mg/L chromium, 0.4 mg/L manganese and 0.3 mg/L molybdenum dissolve. During the ECM-B process, these values are 89.3 mg/L for iron, 2.3 mg/L for chromium, 0.7 mg/L for manganese and 0.5 mg/L for molybdenum. In general, an increased concentration of all investigated elements was determined with increasing current density.

The concentration of the dissolved individual elements chromium, manganese and molybdenum in the ECM electrolyte were placed in a ratio against the dissolved iron to compare both ECM processes (Table 4). If these ratios are compared to the ratio of the elements in bulk material 42CrMo4, it can be seen that the electrolyte is enriched by about twice the amount of chromium and molybdenum compared to the bulk material. However, an enrichment with manganese could not be detected. Molybdenum and chromium are thus dissolved relatively more strongly than iron and manganese in both ECM processes investigated here. A significant influence of the current density on the element ratio in the ECM electrolyte cannot be proven, though.

In addition to the analysis of the material removal rate and the element specific anodic dissolution, the focus of this work is the investigation of the formation, composition and structure of the oxidic rim zone of the martensitic 42CrMo4. The oxide layers are in the range of about <50 nm, and are therefore not visible on SEM cross sections. Even higher-resolution investigations by TEM are not sufficient for a full characterization of the oxide layer on 42CrMo4 steel [10]. Therefore, three different rim zone conditions were examined using XPS: one ground surface before ECM with a natural oxide layer and one surface each after the ECM-A and ECM-B process. The elements oxygen, carbon, iron, chromium and molybdenum were detected qualitatively and quantitatively. Manganese and nitrogen were only qualitatively detected and could not be detected qualitatively due to strong background noise.

It is well known that oxygen and carbon form contamination products at surfaces that are not related to the formation of surface layers during ECM. Therefore, the absolute contents of all measured elements provide only limited information about the actual composition of the rim zones. However, considering the ratio of iron to chromium and of iron to molybdenum allows a more precise evaluation of the XPS results. Table 5 reveals that all three investigated rim zones, including the ground and ECM-A and ECM-B processed rim zones, are enriched with chromium and molybdenum compared to the overall composition of the bulk material 42CrMo4. This enrichment is more pronounced for the ECM rim zones than for the ground rim zone. However, a significant influence of the current density during ECM on the element ratios could not be identified.

**Figure 3:**
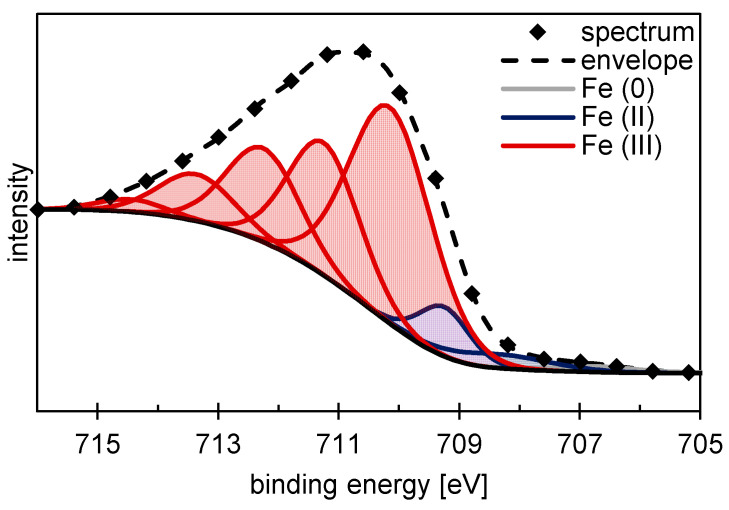
XPS fitting of the Fe 2p_3/2_ region for ECM-B 42CrMo4.

In addition to the content of the different elements, the XPS measurements also provide information about the stoichiometric composition and structure of the formed oxides at the surfaces. Iron is by far the most important metallic component in the material and is therefore a particular focus of attention. According to common models from previous research, the structure Fe_3−x_O_4_ is assumed to be simplified for the oxide layer. X is the variable iron deficiency. According to Biesinger et al. [41], the measured graph of the Fe 2p_3/2_ region, which is shown as an example for the ECM-B surface in Figure 3, can thus be fragmented into the components metallic iron Fe (0), divalent iron Fe (II) and trivalent iron Fe (III), as demonstrated in Table 6.

**Figure 4:**
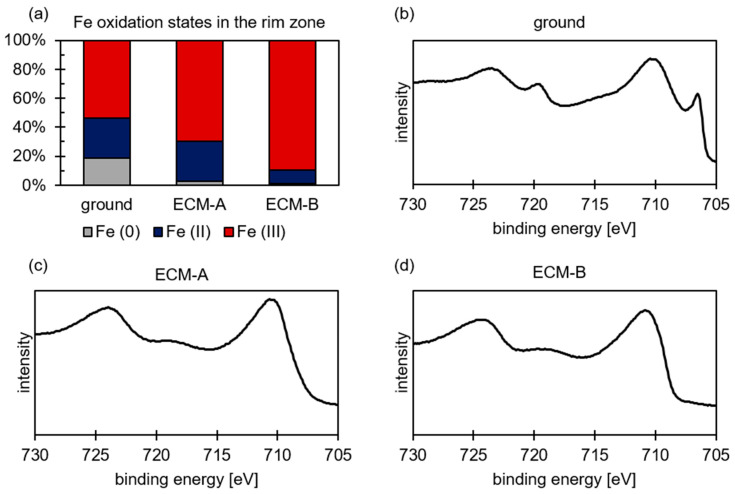
(**a**) Proportion of oxidation states of iron and XPS spectra for (**b**) ground, (**c**) ECM-A and (**d**) ECM-B 42CrMo4 steel.

The evaluation of the XPS results of the ground 42CrMo4 surface indicates that the examined area of the rim zone exhibits a metallic content of about 20% (Figure 4). Approximately 25% is two-valent iron and 55% is three-valent. In contrast to the ground surface, almost no metallic iron can be detected in the two ECM rim zones. The detection of metallic iron for ECM-A and ECM-B is assumed to be within the fluctuation of the measurements and not considered for the further discussion. Furthermore, an influence of the current density during ECM on the oxidation states of iron was also demonstrated. The proportion of trivalent iron increases in the ECM-B process compared to the ECM-A process. As the current density increases, the amount of divalent iron in the oxide layer decreases.

**Figure 5:**
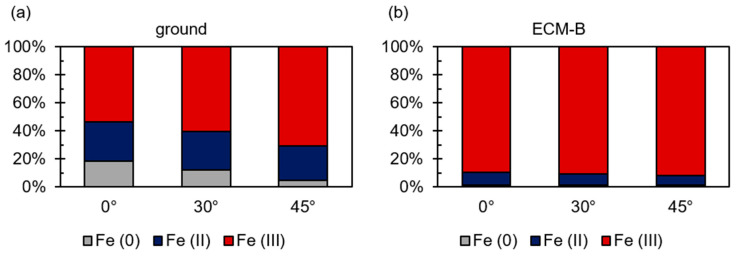
Angle-resolved XPS measurements to determine the oxidation states of iron for (**a**) ground and (**b**) ECM-B 42CrMo4 steel.

Angle-resolved XPS measurements (ARXPS) were performed on one hand to investigate the local differences in the oxidation states of iron in the depth profile, and on the other to evaluate the thickness of the oxides for ground and ECM-B 42CrMo4 (Figure 5). It was observed that the proportion of metallic iron in the ground samples decreases with increasing angle of measurement. Therefore, the proportion of metallic iron decreases towards the oxide surface. Considering the investigations of Ghods et al. [42] on ARXPS measurements of steel, a thickness of 5 to 10 nm of the natural oxide layer of ground 42CrMo4 was estimated. The determination of the oxide thickness of ECM-A and ECM-B 42CrMo4 was estimated to be >10 nm. The exact determination of the thickness was not possible due to the limiting measurement parameters using ARXPS, e.g., minimum and maximum angle. However, the ARXPS measurements also revealed a change in the oxidation state from Fe (II) to Fe (III) for all investigated surfaces within the depth profile. The small changes in the oxidation state dependent on the angle indicate the formation of a mixed iron oxide Fe_3−x_O_4_ for both investigated surfaces. However, the results indicate that the average oxidation number of iron increases more significant from the metal/oxide interface to the oxide/air interface for the native oxide in comparison to the oxide formed during ECM-B processing.

**Figure 6:**
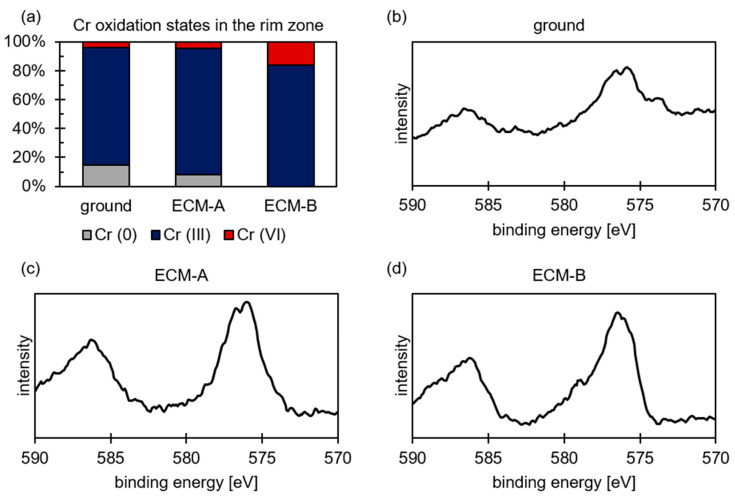
(**a**) Oxidation states of chromium, and XPS spectra for (**b**) ground, (**c**) ECM-A and (**d**) ECM-B 42CrMo4 steel.

Chromium was analyzed in addition to iron using XPS. The XPS fitting parameters for chromium were taken from Biesinger et al. [41]. The results of the XPS measurements reveal that chromium shows almost the same qualitative results as those for iron. As Table 7 and Figure 6 reveal for the Cr 2p_3/2_ region, a significant metallic content was detected in the ground surfaces. This was significantly lower for the ECM surfaces and was related to the fluctuation of the measurements, and was therefore neglected. It is also noticeable that the proportion of hexavalent chromium in the oxide on the surface increases with increasing current density compared to the predominant trivalent chromium.

In addition, molybdenum was investigated by XPS. However, due to the small amount of molybdenum present in the native and the oxide formed after ECM only qualitatively conclusions can be drawn. An increase in the average oxidation number with increasing current density from the ground samples via ECM-A to ECM-B are determined and it is assumed that this is related to the transition from tetravalent to hexavalent molybdenum. Fitting parameters for molybdenum are taken from Baltrusaitis et al. [43].

**Figure 7:**
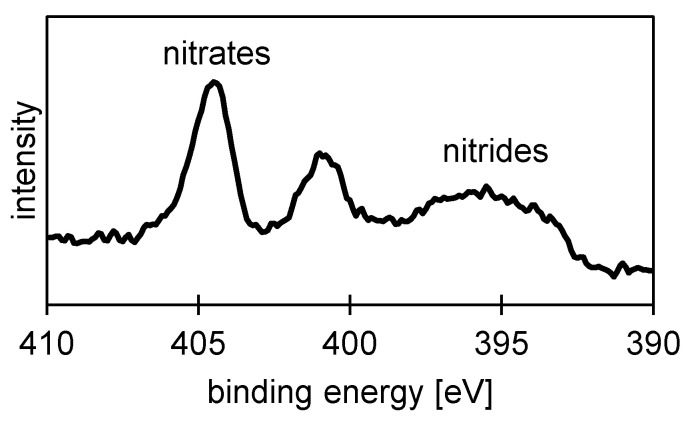
N 1s XPS spectrum of ECM-B 42CrMo4 steel.

Considering the current research and the controversy when discussing the formation of a nitrate-based polishing film during ECM in sodium nitrate solutions, XPS measurements were also performed to obtain detailed results on the occurrence of such a film. The XPS measurements (Figure 7) qualitatively indicate the formation of such a film. Different nitrogen compounds were only found on the ECM surface with the highest current density (ECM-B), such as metal nitrates and metal nitrides [44,45]. No indication of the formation for a nitrate-based polishing film was found for the ECM-A process. It is assumed that based on the small amount of nitrogen components found for the ECM-B process only some residuals of the polishing film were observed.

## 4. Discussion

It is widely known that the oxide formation during transpassive material removal of martensitic 42CrMo4 steel by electrochemical machining significantly influences the material removal rate. However, discussed surrounding the formation, stoichiometric composition and structure of the formed oxides remain controversial. The aim of this work is to investigate how electrochemical machining affects the composition, specifically the alloying element distribution, and structure, given by the oxidation state, of the oxide layer on a martensitic 42CrMo4 steel. In addition, the influence of the current density on the mechanism of oxide layer formation will be discussed.

The XPS and ARXPS investigations provided information on both the chemical composition and the structure of the oxides of the ground and ECM 42CrMo4 rim zones. Considering first the chemical composition, regarding the distribution of the alloying elements within the oxides, the results revealed (Table 5) that both the natural oxide of the ground 42CrMo4 steel and the oxides on the ECM-A and ECM-B surfaces consist of iron oxide enriched with the elements chromium and molybdenum. This enrichment is more pronounced for the oxides after ECM processing compared to the natural oxide formed in air. The proportion of chromium and molybdenum in relation to iron is about 2 to 2.5 times higher after ECM processing in comparison to the native oxide and about 4 to 5 times higher in comparison to the bulk material 42CrMo4. However, an accumulation of manganese could not be observed due to the limitations of the XPS measurements. According to the XANES measurements of the oxide composition of Fe-Cr alloys after polarization at low transpassive potentials of Oblonsky et al. [46], the enrichment of the alloying elements chromium and molybdenum in the oxide formed during ECM at high potentials is attributed to the reduced dissolution rate of these elements compared to iron.

The influence of the current density of the ECM process on the alloying element distribution within the oxides formed at 20.6 A/cm^2^ or 34.0 A/m^2^ was evaluated by the iron/alloying element ratio, considering chromium and molybdenum. No influence of the current density was observed within the context of this work. However, this does not explicitly mean that the local stoichiometric chemical composition of the oxide layer does not change with increasing current density. For example, the oxygen content in the layers could not be measured quantitatively because of impurities at the surface. Therefore, it is possible that the proportion of the elements iron, chromium and molybdenum in the oxide layer decreases compared to oxygen. Such a change would be indicated by a change in the oxidation numbers of the elements.

As a result, the XPS results are discussed in the following according to structural changes indicated by the change of the oxidation state. As demonstrated in Figure 4 and Figure 6, the average oxidation numbers of iron and chromium increase with increasing current density during ECM. A similar effect was observed for molybdenum. This indicates that the oxygen content in the oxide layer also increases. Consequently, it is assumed that the structure and therefore the local stoichiometric chemical composition of the oxide layer changes. With increasing current density, the oxide layer is stoichiometrically transformed from a Fe_3_O_4_-like structure to a Fe_2_O_3_-like structure, considering a Fe_3−x_O_4_ mixed oxide. Evaluating the XPS measurements, it is important to consider that the exact determination of the proportions of the oxidation states of iron and chromium in the oxide layer using XPS is also subject to error. The individual peaks of the oxidation states overlap strongly, as shown exemplarily for iron in Figure 3. Even small shifts in the measured values can lead to a strong change in the Fe (II) to Fe (III) ratio. However, this does not fundamentally challenge the validity of the results. An increase in the oxidation state with increasing current density could be determined qualitatively reliably and independently of each other for iron, chromium and molybdenum. Only the exact proportions of the respective oxidation states are subject to a certain error.

Changes in the oxidation numbers of metals in electrochemically formed oxide layers have already been described in previous investigations. Keller [47] was able to establish a correlation between the applied potential and the oxidation states in oxide layers for iron–chromium alloys in sulfuric acid. At the transition from the passive to the transpassive range, the proportion of Fe (III) increases with increasing potential compared to Fe (II). Lohrengel et al. [27,28,29,30] investigated the influence of the current density on the oxidation states of iron in the transpassive oxide layer by analyzing the amount of Fe (II) and Fe (III) species in the electrolyte by UV-VIS. An increase in the Fe_3_O_4_ content in the transpassive oxide layer was assumed with increasing current density and a decrease of the Fe_2_O_3_ content accordingly. In contrast, Münninghoff [32] could not detect any change in the Fe (II) to Fe (III) ratio as a function of current density during ECM processing of iron in sodium nitrate solution using a microflow cell. However, these investigations on pure iron in sodium nitrate solution using a microflow cell contradict the results presented in this paper for 42CrMo4 after ECM processing.

An explanation for the contradictory results may relate to the fundamentally different experimental setups. Lohrengel et al. [27,28,29,30], as well as Münninghoff [32], worked with a microflow cell. In contrast, an industrial ECM system was used in this work. In particular, the electrolyte flow conditions during the investigations are not comparable, and this could influence the electrochemical transport mechanisms at the surfaces significantly. Lohrengel et al. [27] assumed that a polishing film of supersaturated iron nitrate was formed at the surface of iron during material removal at transpassive potentials. It was assumed that this polishing film became thicker with increasing current densities and hindered the water and oxygen transport to the surface by diffusion. Hence, the oxidation of iron was still thermodynamically possible, but was speed limited by the kinetics. For this reason, it was assumed that the amount of Fe_3_O_4_ on the surface of iron increased with increasing current densities.

The XPS measurements of 42CrMo4 after ECM processing revealed, in addition to the metallic elements iron, chromium and molybdenum, nitrogen, too, and indicated the existence of a polishing film of supersaturated iron nitrate also for 42CrMo4. In the case of the ground surfaces and the ECM-A surfaces, the observed nitrogen was most likely due to contamination. On the surfaces from the ECM process with a high current density of 34.0 A/cm^2^ (ECM-B), however, nitrates and nitrides were detected. The nitrates may have been drying residues of the electrolyte used. However, it is more likely that the observed nitrates and nitrides were formed during the reaction of the electrolyte with the metallic material at high current densities. Datta et al. [26] were also able to detect nitrogen in the oxide layer by XPS investigations on iron, which was dissolved transpassively in sodium nitrate solution. According to Datta et al. [26] the nitrogen originated from the electrolyte and was present in the passive layer in reduced form (N^3−^). It was assumed that the nitrogen penetrated the oxide layer at defects.

The observed change in the oxidation mechanism for 42CrMo4 in comparison to iron reported by Lohrengel et al. [27,28,29,30] and Münninghoff [32] is assumed to relate strongly to a change of the formation of the water-soluble polishing film during ECM processing. Considering a less pronounced water-soluble polishing film of iron nitrate, a change of the transport mechanism towards faster diffusion or even convection, and therefore a strong influence on the oxidation states, is assumed. This may explain the increased oxidation number from Fe (II) to Fe (III) within the ECM oxide layer determined in this study.

Furthermore, the impact of the additional alloying elements, which revealed various oxidation states, such as Cr (III), Cr (VI), Mo (IV) and Mo (VI), must also be considered regarding the change of the oxidation mechanism dependent on the different current densities. Similar to the results for iron, the studies of the oxidation states of the additional alloying elements revealed an increase in the oxidation state with increasing current density due to a higher driving force for oxidation at higher current densities. In addition, the substitution of the different additional alloying elements led to a change in the number of vacancies, as well as to a local change of the stoichiometry. This was driven by the thermodynamics of oxide formation as well as by the oxidation state of the specific species at high transpassive potentials and current densities. Therefore, in addition to the influence of the water-soluble polishing film, the impact of the substitution of the additional elements within the Fe_3−x_O_4_ mixed oxide also has to be considered in order to explain the mechanistic change of oxidation for 42CrMo4 in comparison to iron.

In addition to the already-presented XPS analyses on the structure of the oxide layer, the process electrolyte was examined by ICP-MS. It was demonstrated that the ratios of iron to chromium and iron to molybdenum in the electrolyte after the ECM processes were similar to the ratios of these elements determined in the oxide after ECM. In both cases, a significant enrichment of the elements chromium and molybdenum compared to iron was found in the electrolyte. This indicates that the transpassive metal dissolution in ECM is a stationary process with two parallel running steps. These steps are determined by oxide formation at the metal surface and dissolution of the transpassive oxide into its components.

## 5. Conclusions

In this study, the influence of electrochemical machining on the composition, specifically the chemical distribution of the alloying elements, and structure, e.g., given by the oxidation state, of the transpassive oxide layer on a martensitic 42CrMo4 steel was investigated. A further focus was on investigating the influence of the current density on the mechanism of oxide layer formation at a high transpassive potential. The mechanism of material removal was analyzed with respect to anodic dissolution and oxide formation in sodium nitrate solution used during ECM processing. By linking the electrolyte composition measured by ICP-MS after ECM and the chemical distribution as well as the oxidation states of the single alloying elements within the native oxide, as well as within the oxides measured by (AR)XPS and formed during ECM at 20.6 and 34.0 A/cm^2^, variables influencing the anodic dissolution and oxide formation of the ECM process could be identified. The following conclusions are drawn:

- The material removal rate of ECM-B at 34.0 A/cm^2^ is, independent of the measuring and calculation method, about twice as high as that of ECM-A, at 20.6 A/cm^2^. The efficiency calculation revealed relatively higher losses for the ECM-A process. Therefore, less energy is available for metal dissolution at low current densities.
- The oxide layers formed during ECM are more strongly enriched with chromium and molybdenum than the native oxide layer on ground 42CrMo4.
- With increasing current density of the ECM process, the average oxidation numbers of iron, chromium and molybdenum increase within the oxide layer due to the higher driving force for oxidation.
- The formation of Fe_3−x_O_4_ mixed oxide is concluded to have increasing oxidation state from the inner interface metal/oxide towards the outer interface oxide/electrolyte, in contradiction to previously reported results for iron. The change of the oxidation is related on one hand to the changed transport mechanism related to the formation of a water-soluble polishing film of iron nitrate and on the other hand to the substitution of the additional alloying elements within the Fe_3−x_O_4_ mixed oxide depended on the current densities.
- The iron/alloying element ratio, and therefore the element distribution, within the Fe_3−x_O_4_ mixed oxide measured by XPS is almost similar to the iron/alloying element ratio detected as a reaction product in the electrolyte by ICP-MS. It is concluded that the material removal rate and the efficiency of ECM processing of 42CrMo4 in sodium nitrate solution is strongly determined by a stationary process with two parallel running steps: 1. Transpassive Fe_3−x_O_4_ mixed oxide formation/repassivation; as well as 2. dissolution of the transpassive oxide at the metal surface.

Further work on the influence of microstructure on ECM processing of 42CrMo4 is underway to investigate, e.g., the differences on transpassive oxide formation for ferritic-pearlitic compared to martensitic microstructure. In addition, it is of interest to study the influence of the transpassively formed oxide layers on the functionality of the machined component, for example the oxidation resistance.

## Figures and Tables

**Table 1:** Chemical composition of 42CrMo4 [8].

Element	wt.%	at.%
C	0.43	1.96
Si	0.26	0.51
Mn	0.74	0.74
P	0.01	0.018
S	<0.001	<0.002
Cr	1.09	1.15
Mo	0.25	0.14
Fe	Bal.	Bal.

**Table 2:** ECM process parameters.

Process	Working Gap [µm]	Feed Rate Cathode [µm/s]	Traverse Path Cathode [µm]	Machining Time [s]
ECM-A	1000	1	100	100
ECM-B	500	6	200	33.3

**Table 3:** Material removal rates during ECM calculated by Faraday’s law, height measurement and ICP-MS.

Process	Idealized Removal Rate Calculated by Faraday’s Law [µm/s]	Removal Rate Calculated by Height Measurement [µm/s]	Removal Rate Calculated by ICP-MS [µm/s]
ECM-A	5.95 ± 0.09	3.54 ± 0.52	4.55 ± 0.04
ECM-B	9.83 ± 0.07	6.68 ± 1.40	8.79 ± 0.66

**Table 4:** Iron/alloying element ratios in the ECM process electrolyte and in the bulk 42CrMo4 material.

Sample	Element Ratio Fe/Cr	Element Ratio Fe/Mn	Element Ratio Fe/Mo
Electrolyte ECM-A	38	127	159
Electrolyte ECM-B	40	129	185
Bulk 42CrMo4	89	131	389

**Table 5:** Iron/alloying element ratios in the rim zone of ground and ECM 42CrMo4 measured by XPS compared to the overall composition of the bulk material 42CrMo4.

Sample	Element Ratio Fe/Cr	Element Ratio Fe/Mo
Ground rim zone	54	194
ECM-A rim zone	18	108
ECM-B rim zone	17	88
Bulk 42CrMo4	89	389

**Table 6:** Fe 2p_3/2_ spectral fitting parameters: binding energy and percentage of total area.

Rim Zone	Compound	Peak 1 [eV]	Area [%]	Peak 2 [eV]	Area [%]	Peak 3 [eV]	Area [%]	Peak 4 [eV]	Area [%]	Peak 5 [eV]	Area [%]
Ground	Fe (0)	706.6	18.4								
	Fe (II) in Fe_3_O_4_	708.4	23.3	709.2	4.4						
	Fe (III) in Fe_3_O_4_	710.2	25.4	711.2	9.4	712.3	10.1	713.4	2.3	714.5	6.7
ECM-A	Fe (0)	706.6	2.5								
	Fe (II) in Fe_3_O_4_	708.4	9.5	709.2	18.1						
	Fe (III) in Fe_3_O_4_	710.2	29.5	711.2	27.3	712.3	7.7	713.4	5.3	714.5	0.3
ECM-B	Fe (0)	706.6	1.2								
	Fe (II) in Fe_3_O_4_	708.4	3.3	709.2	5.9						
	Fe (III) in Fe_3_O_4_	710.2	41.3	711.2	22.0	712.3	16.1	713.4	8.2	714.5	2.0

**Table 7:** Cr 2p_3/2_ spectral fitting parameters: binding energy and percentage of total area.

Rim Zone	Compound	Peak 1 [eV]	Area [%]	Peak 2 [eV]	Area [%]	Peak 3 [eV]	Area [%]	Peak 4 [eV]	Area [%]	Peak 5 [eV]	Area [%]
Ground	Cr (0)	574.2	14.8								
	Cr (III)	575.7	34.2	576.7	21.5	577.5	17.7	578.5	6.3	578.9	1.5
	Cr (VI)	579.6	4.2								
ECM-A	Cr (0)	574.2	8.4								
	Cr (III)	575.7	32.0	576.7	29.4	577.5	20.0	578.5	4.5	578.9	1.3
	Cr (VI)	579.6	4.4								
ECM-B	Cr (0)	574.2	0.0								
	Cr (III)	575.7	27.5	576.7	28.4	577.5	19.0	578.5	6.2	578.9	2.7
	Cr (VI)	579.6	16.3								

## Data Availability

The data presented in this study are available on request from the corresponding author.
